# Hypoxia-inducible factor-1α and vascular endothelial growth factor expression in circulating tumor cells of breast cancer patients

**DOI:** 10.1186/bcr2452

**Published:** 2009-11-17

**Authors:** Galatea Kallergi, Harris Markomanolaki, Vicky Giannoukaraki, Maria A Papadaki, Areti Strati, Evi S Lianidou, Vassilis Georgoulias, Dimitris Mavroudis, Sofia Agelaki

**Affiliations:** 1 Laboratory of Tumor Cell Biology, School of Medicine, University of Crete, Voutes, Heraklion, 71110, Greece; 2 Laboratory of Analytical Chemistry, Department of Chemistry, University of Athens, 15771, Greece; 3 Department of Medical Oncology, University General Hospital of Heraklion, Voutes, Heraklion, 71110, Greece

## Abstract

**Introduction:**

The detection of peripheral blood circulating tumor cells (CTCs) and bone marrow disseminated tumor cells (DTCs) in breast cancer patients is associated with a high incidence of disease relapse and disease-related death. Since hypoxia-inducible factor-1α (HIF-1α) and vascular endothelial growth factor (VEGF) play an important role in angiogenesis and tumor progression, the purpose of the current study was to investigate their expression in CTCs.

**Methods:**

The expression of cytokeratins (CK), VEGF, vascular endothelial growth factor receptor-2 (VEGF2), HIF-1α and phosphorylated-focal adhesion kinase (pFAK) in CTCs from 34 patients with metastatic breast cancer who had detectable CK-19 mRNA-positive CTCs was assessed using double staining experiments and confocal laser scanning microscopy. Peripheral blood mononuclear cells (PBMCs) were stained with a monoclonal A45-B/B3 pancytokeratin antibody in combination with either VEGF or VEGFR2 or HIF-1α or pFAK antibodies, respectively.

**Results:**

pFAK expression in circulating tumor cells was detected in 92% of patients whereas expression of VEGF, VEGF2 and HIF-1α was observed in 62%, 47% and 76% of patients, respectively. VEGF, VEGF2, HIF-1α and pFAK were expressed in 73%, 71%, 56% and 81%, respectively, of all the detected CTCs. Vascular endothelial growth mRNA was also detected by quantitative real-time RT-PCR in immunomagnetically-separated CTCs. Double and triple staining experiments in cytospins of immunomagnetically-isolated CTCs showed that VEGF co-expressed with HIF-1α and VEGF2.

**Conclusions:**

The expression of pFAK, HIF-1α, VEGF and VEGF2 in CTCs of patients with metastatic breast cancer could explain the metastatic potential of these cells and may provide a therapeutic target for their elimination.

## Introduction

The development of metastasis is mainly responsible for cancer-related death. Malignant cells detached from the primary tumor possessing advantageous biological characteristics are presumed to generate distant disease sites. Indeed, it has been shown that metastasis is associated with the presence of circulating (CTCs) and disseminated (DTCs) tumor cells in peripheral blood and bone marrow, respectively, of otherwise metastasis-free patients [1,2]. Several studies have reported that the detection of CTCs and DTCs represents a strong and independent prognostic factor for a decreased disease-free and overall survival [3-5]. In addition, recent studies have shown that in patients with metastatic breast cancer, the assessment of CTCs is an earlier and more reliable marker than that of DTCs, associated with disease prognosis and suitable for monitoring of tumor response to chemotherapy [6].

Angiogenesis, primarily regulated by vascular endothelial growth factor (VEGF), is a critical event in tumor progression and metastasis [7]. Tumor cells produce and release vascular endothelial growth factor in response to oxygen and nutrients deprivation that in turn stimulates the formation of new vessels and promotes tumor growth and dissemination [8]. Besides its role in new blood formation, VEGF has been also shown to stimulate the proliferation of tumor cells. Indeed, VEGF enhanced the proliferation and the migration of breast cancer cells [9,10], whereas siRNA targeting of VEGF in MCF7 breast cancer cell line, effectively inhibited cell proliferation and enhanced apoptosis [11]. Moreover, VEGF expression in breast cancer cells was correlated with decreased response to hormone treatment [9] and with reduced survival in breast cancer patients [12]. Focal adhesion kinase (FAK) and Src catalytic activities are important in promoting VEGF-induced tumor angiogenesis [13] whereas the inhibition of FAK reduces VEGF expression resulting in small avascular tumors in mice [14].

VEGFR2 is the main receptor for VEGFs and its action is related to the activation of signalling molecules such as PLCγ1, phosphoinositide-3 kinase (PI-3 kinase), Akt, Src and ERK [15].

The expression of VEGF in cancer cells is under the control of hypoxia-inducible factor-1α (HIF-1α) which is a transcription factor induced under hypoxia conditions [16]. In the absence of oxygen, HIF-1α binds to hypoxia-response elements inducing the transcription of VEGF gene among other factors such as LDH-A, NOS, EPO and so on [16]. The expression of HIF-1α depends on FAK and phosphoinositide-3 kinase (PI-3) activation in cancer cells [17].

Our group has recently demonstrated that FAK as well as PI-3 and Akt kinases are phosphorylated and thus activated in CTCs of breast cancer patients [18,19]. Since FAK is implicated in the angiogenesis process and induces the expression of VEGF, it was of interest to evaluate whether CTCs from breast cancer patients have activated the angiogenesis pathway by expressing HIF-1α and VEGF. This could be an important mechanism associated with the metastatic potential of these cells and therefore could bear important therapeutic implications.

## Materials and methods

### Cell cultures

All cell lines were obtained from ATCC (American Type Culture Collection, USA). The MCF7 mammary adenocarcinoma cells were cultured in 1:1 (v/v) Dulbecco's Modified Eagle (DMEM)/Ham's F12 medium (GIBCO-BRL NY, USA), supplemented with 10% fetal bovine serum (FBS), 2 mM L-glutamine, 30 mM NaHCOB_3B_, 16 ng/ml insulin and 50 mg/ml penicillin/streptomycin. MDA-MB-231 and human umbilical vein endothelial cells (HUVEC) cells were cultured in DMEM 10% FBS whereas the MDA-MB-453, SKBR3 and T47D cells were cultured in RPMI 10% FBS. Subcultivation of all cell lines was performed with 0.25% trypsin and 5 mM EDTA. These cell lines were used as positive controls for VEGF, vascular endothelial growth factor receptor 2, HIF-1α and phosphorylated-focal adhesion kinase (pFAK) expression. Cells were maintained in a humidified atmosphere of 5% COB_2B _in air. All experiments were performed during the logarithmic growth phase of the cells. Fifteen to 20 h prior to the experiments, cells were transferred to serum-free culture medium containing only L-glutamine, NaHCOB_3B _and penicillin/streptomycin. HUVEC cells were used as a positive control for HIF-1α expression [20].

### Patient samples and cytospin preparation

Blood samples obtained before the initiation of the respective line of treatment from 34 patients with metastatic breast cancer and tumor cells circulating in their peripheral blood as detected by real-time PCR for Cytokeratin (CK) -19 mRNA [21], were analyzed. Peripheral blood (10 ml in EDTA) was drawn from the middle of vein puncture after the first 5 ml of blood were discarded. This precaution was undertaken in order to avoid contamination of the sample with epithelial cells from the skin during sample collection. All patients gave their informed consent to participate in the study, which has been approved by the Ethics and Scientific Committees of our institution.

Peripheral blood mononuclear cells (PBMC) were isolated with Ficoll-Hypaque density gradient (d = 1,077 g/mol) centrifugation at 1,800 rpm for 30 minutes. PBMCs were washed three times with Phosphate Buffered Saline solution (PBS) and centrifuged at 1,500 rpm for 10 minutes. Aliquots of 250,000 cells were centrifuged at 2,000 rpm for two minutes on glass slides. Cytospins were dried up and stored at -80°C. Four to five slides were evaluated from the same blood sample were evaluated for each examined molecule in the corresponding double staining experiments. The results were expressed as the mean number of CTCs/250,000 PBMCs.

### Confocal laser scanning microscopy

The presence of CK-positive cells in PBMCs of cytospin preparations was investigated using the A45-B/B3 (Micromet, Munich, Germany) mouse antibody (detecting CK8, CK18 and CK19). Cytospins were also double stained with anti-CD45 (common leukocyte antigen) antibody to exclude possible illegitimate expression of cytokeratins on hematopoietic cells. The cytomorphological criteria (high nuclear to cytoplasmic ratio, larger than white blood cells and so on) proposed by Meng *et al *[22] were used in order to characterize a CK-positive cell as a CTC. Cytospins from the same patients were also stained with goat anti-VEGF (R&D Systems, Inc, Minneapolis, USA), rabbit anti-VEGFR2 (Cell Signaling, Boston, USA), rabbit anti-HIF-1α (Santa Cruz, Santa Cruz, CA, USA) and mouse anti-pFAK (Chemicon, Temecula, CA, USA) antibodies in double staining experiments and the expression of these molecules was assessed using confocal laser scanning microscopy [18] (see additional data file 1). Double staining experiments validating VEGF, VEGFR2 and HIF-1α expression in 10 healthy blood donors were also performed as controls. Control experiments for pFAK expression have been previously described [18].

For double staining experiments PBMC cytospins were fixed with 3% paraformaldehyde (PFA) for 30 minutes. Cell membrane permeabilization was performed with 0.5% Triton for 10 minutes followed by overnight incubation with blocking buffer (PBS/1%BSA). Subsequently, slides were stained for cytokeratin with the A45-B/B3 antibody and for VEGF, VEGFR2 or HIF-1α with the corresponding antibodies for 45 minutes. Cells were then incubated with the appropriate secondary antibody for one hour. Finally, slides were stained with DAPI conjugated with antifade (Invitrogen, Carlsbad, CA, USA). Phospho-FAK (pFAK) staining was performed as previously described [18]. Samples were analyzed using a confocal laser scanning microscope module (Leica Lasertechnik, Heidelberg, Germany) and images were analyzed with the respective software.

Triple immunofluoresence for CK/VEGF/HIF-1α and CK/VEGF/VEGFR2 was also performed. Cells were initially fixed using 4% formaldehyde for 15 minutes at room temperature (RT). Permeabilization was achieved with 0.1% Triton X-100 for five minutes at RT. After blocking with PBS supplemented with 10% (v/v) FBS for 30 minutes, cells were incubated with the corresponding antibodies for 45 minutes each. Zenon technology (Fluorescein Isothiocyanate (FITC)-conjugated IGg1 antibody) (Molecular Probes, Invitrogen, Carlsbad, CA, USA) was used for CK detection with the A45-B/B3 antibody. Zenon antibodies were prepared within 30 min before use. Zenon reagent was incubated with A45-B/B3 for five minutes, blocking buffer was added for 5 min and the conjugated antibodies were then ready for use.

VEGFR2 was detected using anti-rabbit antibody labelled with Alexa555 (Molecular Probes, Invitrogen, Carlsbad, CA, USA), whereas VEGF was detected by anti-mouse antibody (Santa Cruz, Santa Cruz, CA, USA) labelled with Alexa 633 (Molecular Probes, Invitrogen, Carlsbad, CA, USA). For CK/VEGF/HIF-1α staining, zenon technology (FITC-conjugated) was also used for CK detection. For VEGF, an anti-mouse antibody labelled with Alexa 633 was used and HIF-1α was stained with anti-rabbit antibody labelled with Alexa555. Cells incubated with the different antibodies were post-fixed with 4% (v/v) formadehyde in PBS for 15 minutes at RT. Finally, cells were stained with DAPI conjugated with antifade.

### Immunomagnetic separation of CTCs

In 10 patients with metastatic breast disease, a negative selection procedure was performed for the isolation of CTCs according to Naume *et al *[23]. Briefly, 100 μl of CELLection beads coated with anti-CD45 monoclonal antibody (Dynal, Invitrogen, Carlsbad, CA, USA) were added in 10^7P^/ml PBMCs in PBS/0.1% BSA/2 mM EDTA. PBMCs were isolated from 20 ml of blood using Ficoll density gradient centrifugation. After an incubation for 30 minutes at 4°C, the supernatant was transferred in FBS-coated tubes and cells were cyto-centrifuged at 2000 rpm for two minutes on glass slides. The same number of cells was centrifuged at 1,500 rpm for five minutes and the pellet was stored at -80°C for RNA extraction.

In control spiking experiments, 10 MCF7 tumor cells (1/10^6^), 100 cells (10/10^6^) and 1,000 cells (100/10^6^) were spiked in 10^7 ^PBMCs obtained from healthy volunteers. Subsequently, 100 μl of anti-CD45-beads were added in every 10^7 ^PBMCs according to the manufacturer's instructions and cytospins were prepared using the isolated cells. By this method, it was possible to estimate the enrichment of CTCs per 250,000 PBMCs. The proportion of CTC/PBMCs after enrichment was approximately 1/60 and the obtained degree of CTCs enrichment ranged from 150% to 300%. Moreover, in order to verify that the tumor cell-depleted PBMCs fraction did not contain CTCs, immunostaining with A45-B/B3 antibody was performed on cytospins prepared from the CTCs-depleted fraction. Three independent spiking experiments demonstrated that no CK-positive cells could be identified in these samples.

### Immunoblot analysis

MCF7, T47D, SKBR3, MDA-MB-453 and MDA-MB-231 cells were lysed in 100 μl lysis buffer each (50 mM Tris/HCl, 1% TritonX-100 pH 7.4, 1% Sodium deoxycholate, 0.1% SDS, 0.15% NaCl, 1 mM EDTA, 1 mM sodium orthovanadate) at 4°C and flasks were scrapped off. The remaining insoluble material was removed by centrifugation. Protein concentration of the samples was determined using the Bio-Rad protein kit. Equal amounts of protein (100 μg) were separated with SDS electrophoresis and blotted onto nitrocellulose membrane. Proteins were incubated with anti-VEGF, anti-VEGFR2 and HIF-1α antibodies for 1 h at room temperature and then with the appropriate secondary antibody. Detection of protein bands was succeeded using the ECL kit. Western blots were quantified using Bio-Rad Quantity One program. The numbers we provided correspond to the Adj Vol Intensity per mm^2 ^and the measurements concerned equal square area. Intensity of the background was set as 0.

### Real-time RT-PCR for VEGF quantification

RNA was extracted, from both the immunomagnetically CTC-enriched and CTC-depleted PBMCs fractions, using the Trizol reagent (Invitrogen, Carlsbad, CA, USA). The same procedure was followed for three normal volunteers. Both fractions were used in order to investigate the relative expression of VEGF since it could not be excluded that some hemopoietic cells had contaminated the CTC-enriched fraction. All preparations and handling of RNA took place in a laminar flow hood under RNase-free conditions. RNA concentration was quantified with Nanodrop ND-1000 spectophotometer. Afterwards equal RNA amounts from patients' CTCs-enriched and CTCs-depleted PBMCs proceeded to cDNA synthesis with Superscript III platinum kit (Invitrogen, Carlsbad, CA, USA) according to manufacturer's instructions. Real-time RT-PCR for VEGF quantification was performed as previously described [24].

## Results

### Patient characteristics

Thirty-four patients with metastatic breast cancer and CK-19 mRNA positive CTCs detected in their peripheral blood [21], were enrolled in the present study. Patient characteristics are listed in Table 1. Median age was 57 (range, 30 to 76) years and 88% of the patients were post-menopausal. Twenty-three (68%) patients had relapsed after treatment for early breast cancer. Adjuvant chemotherapy regimens included anthracyclines in four patients, taxanes and anthracyclines in 10, Cyclophosphamide, Methotrexate and 5-Fluoruracil (CMF regimen) in six patients, whereas in three the type of adjuvant chemotherapy was unknown. Eighteen (53%) patients had received no prior chemotherapy for metastatic disease, seven (21%) had received one and nine (26%) two or more prior lines of treatment. Patients had not received antiangiogenic agents as part of their treatment before study entry. Patients with HER2 positive tumors received chemotherapy in combination with trastuzumab or lapatinib. Second- and third- (or higher) line regimens administered included anthracyclines or taxanes as single agents or combinations of docetaxel and capecitabine, taxanes and antracyclines, vinorelbine and gemcitabine and vinorelbine in combination with trastuzumab or lapatinib in patients with HER2 positive tumors.

**Table 1:** Patient characteristics

**Age**	
Median (range)	57 (30 to 76)
	
	**n (%)**
	
**Menopausal status**	
Premenopausal	4 (12)
Postmenopausal	30 (88)
**Primary breast cancer at presentation**	23 (68)
**Tumor size (cm)**	
1-1.9/2-4.9/≥5/UN	3 (13)/14 (61)/2 (9)/4 (17)
**Lymph nodes**	
0/1-3/≥4/UN	7 (30)/6 (26)/8 (35)/2 (9)
**Grade**	
II/III/UN	8 (35)/11 (48)/4 (17)
Disease sites*	
1	5 (15)
2	15 (44)
3	5 (15)
≥4	8 (23)
Unknown	1 (3)
**Predominantly visceral disease**	
Yes	11 (32)
No	22 (64)
Unknown	1 (3)
Line of treatment **	
0	18 (53)
1	7 (21)
≥2	9 (26)
**ER/PR status**	
Positive	16 (47)
Negative	13 (38)
Unknown	5 (15)
**HER2 status**	
Positive***	7 (20)
Negative	23 (68)
Unknown	4 (12)

UN = unknown,

* Numbers correspond to the number of organs involved

** Numbers correspond to the number of prior therapies administered for metastatic disease

*** IHC:3+ or FISH positive

### VEGF and VEGFR2 expression in breast cancer cell lines

**Figure 1:**
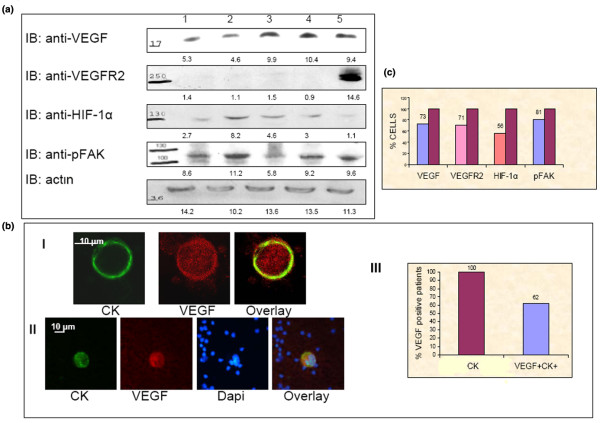
Vascular endothelial growth factor expression in breast cancer cell lines and in circulating tumor cells. A: VEGF, VEGFR2 HIF-1α and pFAK expression in cultured breast cancer cell lines, 1: MCF7, 2:T47D, 3: SKBR3 4:MDA MB-453, 5: MDA MB-231 (western blot analysis of equal protein amount of cell lysates). B (I): Representative images of confocal laser scanning microscopy of PBMCs cytospins stained with pancytokeratins A45 B/B3 (green), and VEGF anti-goat (red) antibodies. B (II): Representative images of PBMCs cytospins stained with pancytokeratins A45 B/B3 (green), VEGF anti-goat (red) antibodies and Dapi (blue). B (III): Quantification of VEGF and CK co-expression in CTCs of 34 metastatic breast cancer patients. All the examined patients (100%) presented CTCs in their blood, while 62% also presented double positive cells (VEGF^+^CK^+^). (C) Quantification of double positive CTCs/total CTCs for each examined molecule in 34 metastatic breast cancer patients. For pFAK testing cytospins from 12 patients were evaluated. indicatively. Seventy-three percent of the examined CTCs were VEGF^+^CK^+ ^positive, 71% were VEGFR2^+^CK^+^, 56% were HIF-1α^+^CK^+ ^and 81% were pFAK^+^CK^+^.

In preliminary experiments, we investigated the expression of VEGF, VEGFR2 HIF-1α and pFAK in breast cancer cell lines. Among the five examined breast cancer cell lines MDA-MB-231, MDA-MB-453 and SKBR3 cells are generally considered more aggressive compared to T47D and MCF7 cells [25-27]. Immunoblot analysis of equal protein amounts of cell lysates with anti-VEGF antibody revealed that the less-aggressive cell lines (T47D and MCF7) expressed less VEGF than the more aggressive cell lines SKBR3, MDA-MB-231 and MDA-MB-453 cells (Figure 1A). HIF-1α and pFAK was expressed in all the examined cell lines except MDA-MB-231, whereas VEGFR2 was mainly expressed in MDA-MB-231 (see additional data file 2).

Subsequently, the sensitivity and specificity of immunofluorescence assays for the detection of VEGF, VEGFR2 and HIF-1α in CTCs were evaluated on cytospins of tumor cells spiked in PBMCs obtained from healthy volunteers.

### VEGF and VEGFR2 expression in CTCs

Using double staining experiments, VEGF-expressing CK-positive CTCs were detected in 21 (62%) patients (Figure 1BI, II, III, see also additional data file 3). Among a total of 99 CK-19 positive CTCs recognized in cytospins from all patients, 72 (73%) stained positively for both VEGF and CK (Figure 1C) showing that the majority of the detected CTCs expressed VEGF in their cytoplasm. Moreover, in 12 (35%) patients all the detected CTCs expressed VEGF in their cytoplasm. The median percentage of CTCs expressing VEGF among the 34 patients was 100% (range; 29% to 100%).

**Figure 2:**
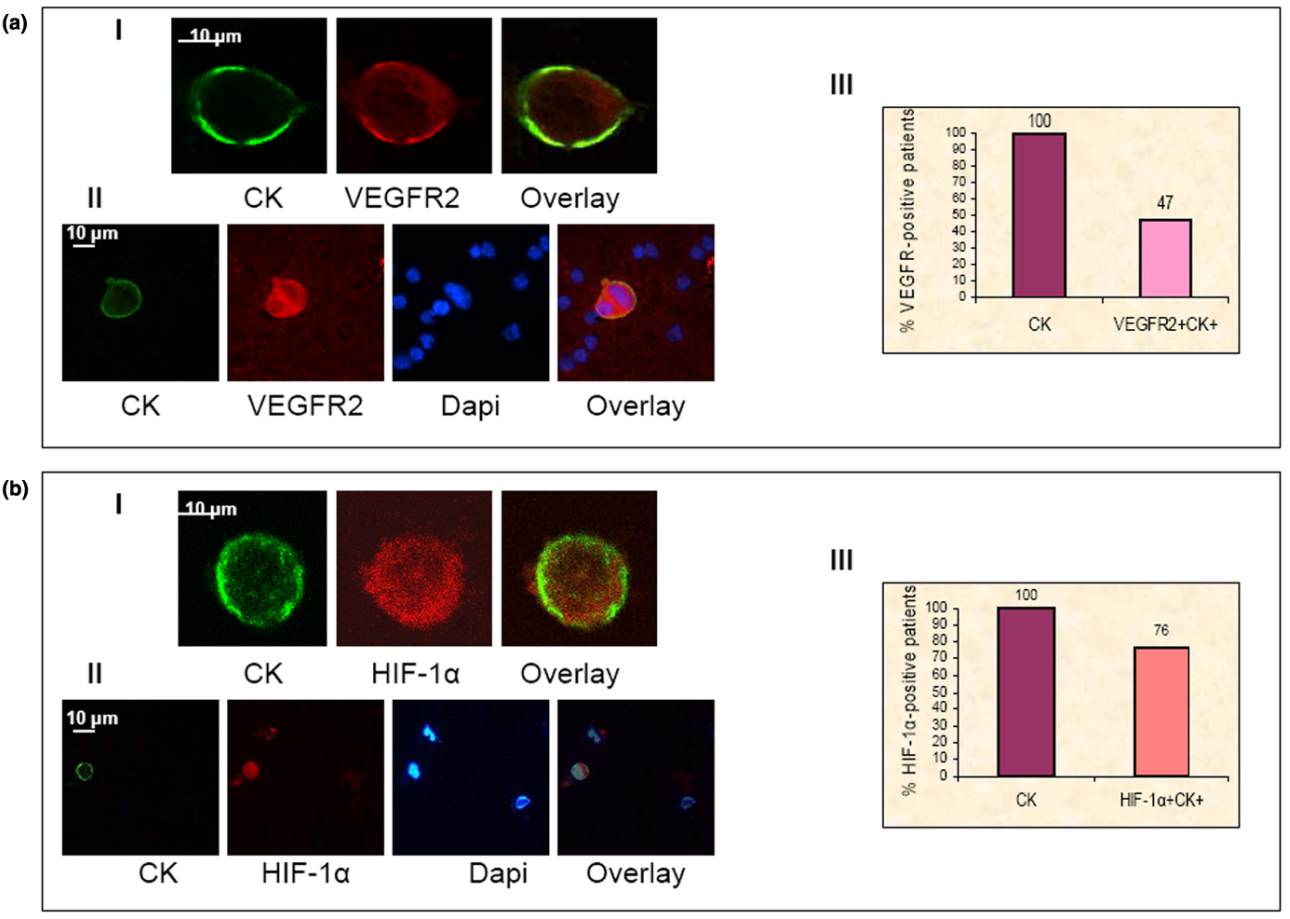
Vascular endothelial growth factor receptor 2 and hypoxia-inducible factor-1α in circulating tumor cells of patients with breast cancer. A (I): Representative images of cytospin double stained with monoclonal pancytokeratin (A45-B/B3) (green) and polyclonal VEGFR2 anti-rabbit (red) antibodies. A (II): Representative images of PBMCs cytospins stained with pancytokeratins A45 B/B3 (green), VEGFR2 anti-rabbit (red) antibodies and Dapi (blue). A (III): Quantification of VEGFR2 and CK co-expression in CTCs of 34 patients with metastatic breast cancer. CTCs were detected in all (100%) patients while in 47% double positive cells (VEGFR2^+^CK^+^) were identified. B (I): Representative image of a cytospin double stained with monoclonal pancytokeratin (A45-B/B3) (green) and polyclonal HIF-1α (red) antibodies. B (II): Representative images of PBMCs cytospins stained with pancytokeratins A45 B/B3 (green), HIF-1α anti-rabbit (red) antibodies and Dapi (blue). B (III): Quantification of HIF-1α and CK co-expression in CTCs of 34 patients with metastatic breast cancer. All the examined patients (100%) harvested CTCs in their blood, while 76% also presented double positive cells (HIF-1α^+^CK^+^).

Consequently, VEGFR2-expressing CK^+ ^CTCs were identified in 16 (47%) out of 34 patients (Figure 2AIII, see also additional data file 3). Among a total of 124 CK^+ ^CTCs detected in all studied patients, 89 (71%) were double stained [(CK(+)/VEGFR2(+)] (Figure 1C). In only four (12%) patients, all the detectable CTCs expressed VEGFR2. The median expression of VEGFR2 on CTCs identified per patient was 75% (range, 10% to 100%). Confocal laser scanning microscopy revealed that VEGFR2 was mainly localized adjacent to the plasma membrane (Figure 2AI); however, in some CTCs, VEGFR2 was also intracellulary distributed (Figures 2AII, 3BI and 3CI). Spearman's analysis showed a significant correlation (*P *= 0.042) between the number of VEGF- and VEGFR2-expressing CTCs in the whole group of patients. No double positive cells (CK^+^VEGF^+ ^or CK^+^VEGFR2^+^) could be observed in healthy volunteers (n = 10 normal blood donors), although VEGF and VEGFR2 expression was detected in some hematopoietic cells.

**Figure 3:**
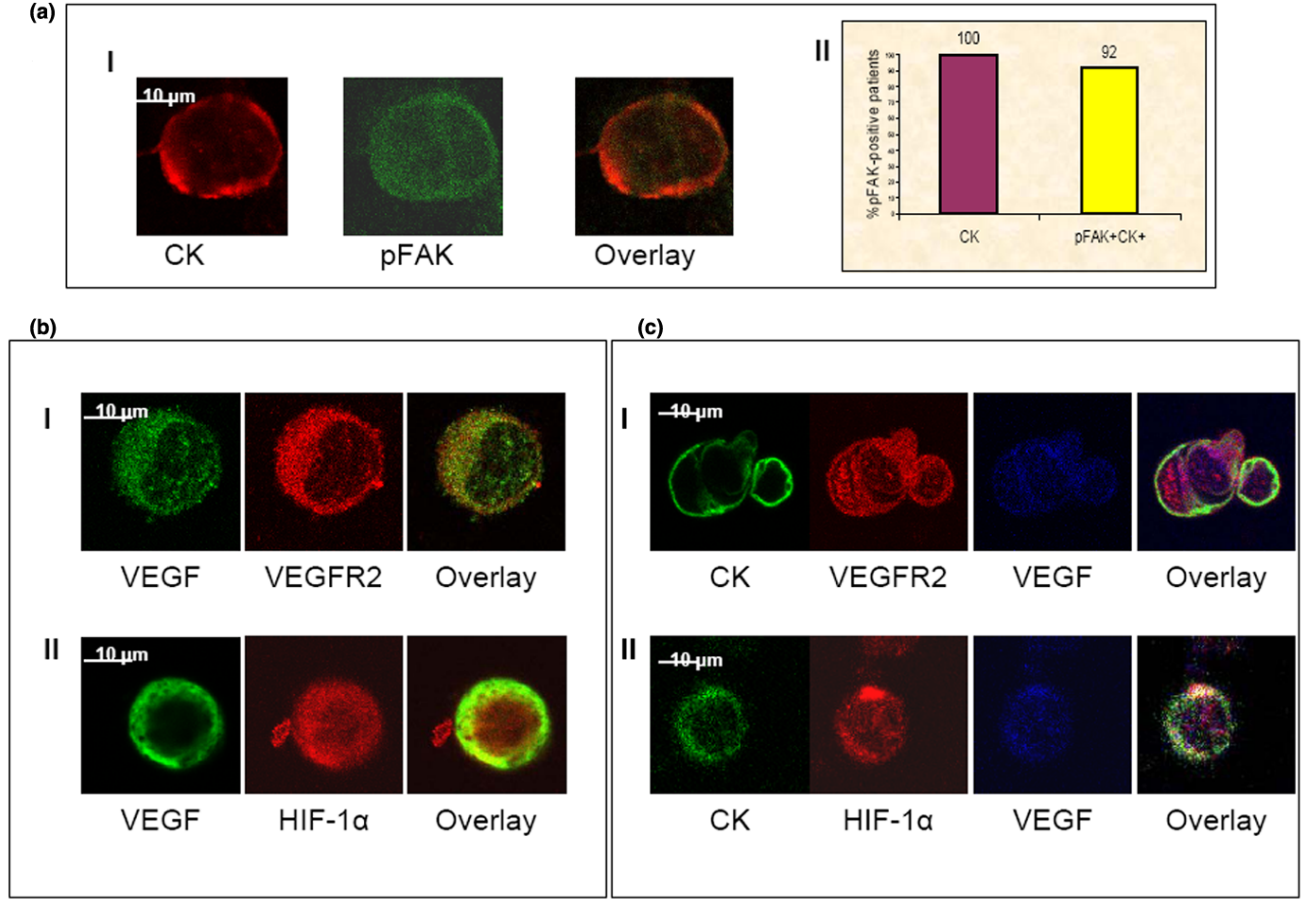
Phosphorylated-focal adhesion kinase expression in circulating tumor cells and co-expression of vascular endothelial growth factor/vascular endothelial growth factor receptor or vascular endothelial growth factor/hypoxia-inducible factor-1α in circulating tumor cells of patients with breast cancer. A (I): Representative image of a cytospin double stained with polyclonal pancytokeratin (red) and (monoclonal) pFAK (green) antibodies. A (II): Quantification of pFAK and CK co-expression in CTCs of 12 patients with metastatic disease. All the examined patients (100%) presented CTCs in their blood, while 92% also presented double positive cells (pFAK^+^CK^+^). B: Representative confocal laser scanning micrographs of CTCs cytospins after negative immunomagnetic separation in 10 patients with metastatic breast cancer. Cells cytospins were double stained with VEGF and either (I) VEGFR2 or (II) HIF-1α antibodies, respectively. C: Representative image of a cytospin, triple stained with monoclonal pancytokeratin (A45-B/B3) (green) and VEGF antibodies (blue) along with either (I) VEGFR2 or (II) HIF-1α (B) (red) polyclonal antibodies (red).

The expression of either VEGF or VEGFR2 on CTCs did not correlate with ER/PR or HER2 status of the primary tumor. In addition, no correlation was found between VEGF and VEGFR2 expression and line of treatment (no prior regimens vs one or more regimens for metastatic disease) or response to treatment.

### Expression of HIF-1α and phosphorylated FAK in CTCs

VEGF expression is under the regulation of HIF-1α transcription factor and of FAK [28]. In order to determine whether angiogenic pathways including HIF-1α and FAK operate on CTCs, the expression of these molecules was assessed using double staining experiments in patient's cytospins.

Figure 2BIII demonstrates that 26 (76%) out of 34 patients had HIF-1α^+^/CK^+^-positive cells. HIF-1α is distributed both in the cytoplasm and in the nucleus of the majority of CTCs (Figure 2BI and 2BII, see also additional data file 3). In nine (26%) patients, all the detected CTCs expressed HIF-1α; the median proportion of cells expressing HIF-1α was 69% (range; 5% to 100%). Among a total of 223 CK-positive cells detected in all studied patients, 125 (56%) were double-positive (CK^+^/HIF-1α^+^) (Figure 1C). HIF-1α-positive CTCs co-exist with VEGF- or VEGFR2-positive CTCs in 16 (47%) and 14 (41%) breast cancer patients, respectively.

Double staining experiments in the control group of 10 healthy blood donors (n = 10), revealed no CK-positive cells expressing HIF-1α in their PBMCs, whereas this transcription factor was expressed in some hematopoietic cells.

We have recently reported that pFAK was expressed in all CK-positive CTCs of patients with early breast cancer [18]. In the present study we investigated the expression of pFAK in 12 patients with metastatic disease for whom adequate material was available. Double staining experiments revealed that pFAK was expressed in 92% (11 out of 12) of the CTCs-positive patients (Figure 3AI and 3AII). Among a total of 26 CK^+ ^CTCs, 21 (81%) were double stained (CK^+^pFAK^+^) (Figure 1C). In eight (67%) out of 12 patients, all their CTCs were pFAK-positive. All of these patients also had HIF-1α-positive CTCs (Spearman's rho correlation analysis *P *< 0.001). The median percentage of cells expressing pFAK was 100% (range 50% to 100%). No correlation was also found between the expression of HIF-1α on CTCs and ER/PR or HER2 status of the tumor as well as with the line of treatment or response to treatment.

**Figure 4:**
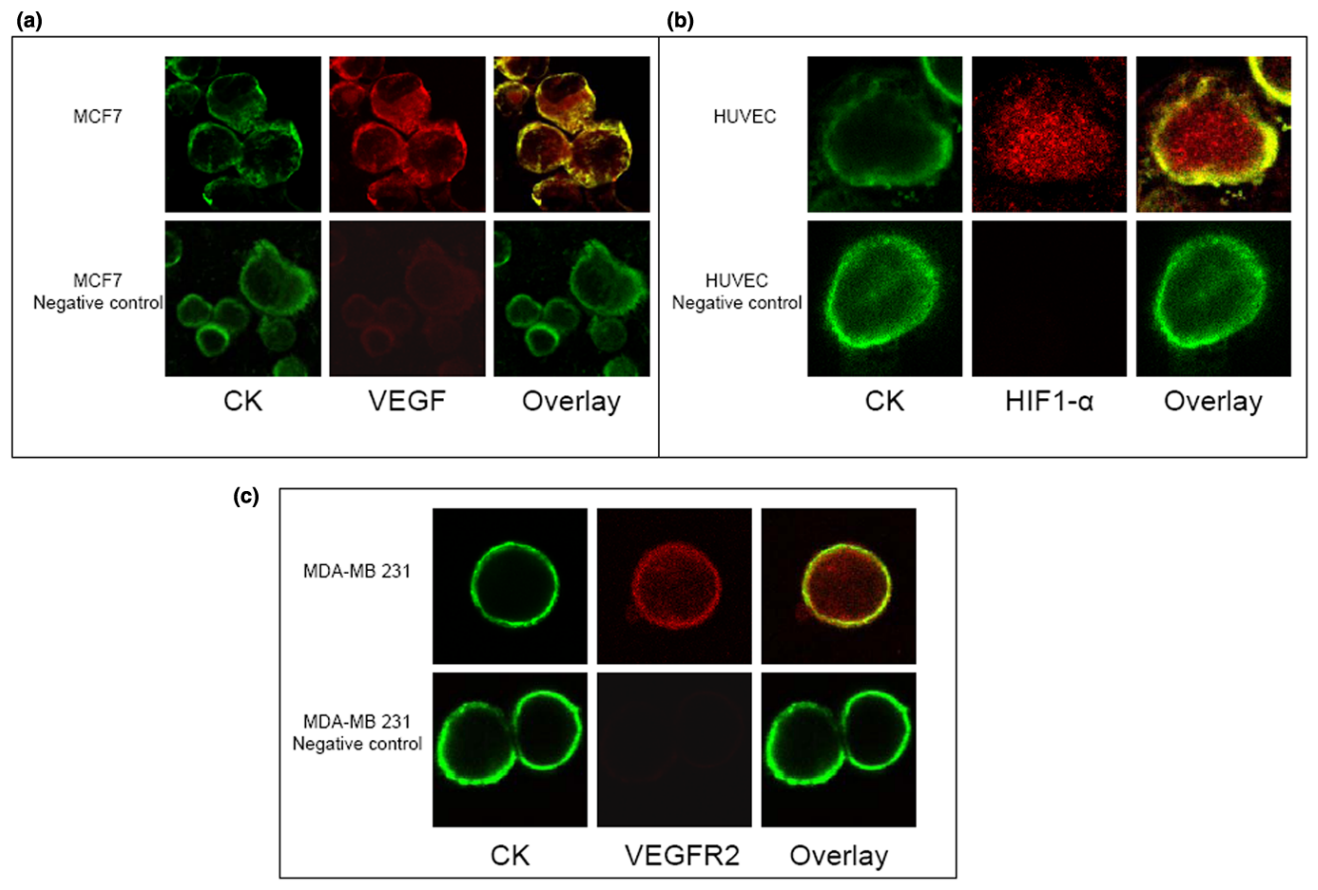
Vascular endothelial growth factor, vascular endothelial growth factor receptor 2 and hypoxia-inducible factor-1α expression in MCF7, Human umbilical vein endothelial cells and MDA-MB-231 respectively. A: MCF7 cells stained with pancytokeratin/FITC (green) and VEGF/Alexa555 (red) or MCF7 cells stained with pancytokeratin/FITC (green) and just Alexa555 antibody without VEGF antibody (negative control). B: HUVEC cells stained with pancytokeratin/FITC (green) and HIF1-α/Alexa555 (red) or HUVEC cells stained with pancytokeratin/FITC (green) and just Alexa555 antibody without HIF1-α antibody (negative control). C: MDA-MB-231 cells stained with pancytokeratin/FITC (green) and VEGFR2/Alexa555 (red) or MDA-MB-231 cells stained with pancytokeratin/FITC (green) and just Alexa555 antibody without VEGFR2 antibody (negative control).

Negative and positive controls were used in each experiment for every examined molecule Figure 4A, B and 4C.

### Co-expression of VEGF, VEGFR2 and HIF-1α in CTCs

The co-expression of the examined molecules was further analysed in 10 patients after negative immmunomagnetic separation. Negative selection was preferred because prior experiments using MCF7 cells spiked into PBMCs obtained from healthy donors to estimate recovery yields, revealed higher recovery rates for negative compared to positive selection, even in low cell concentrations (one CTC in 10^6 ^PBMCs); moreover, the quality of RNA (ratio 260:280) extracted from CTCs-enriched and CTCs-depleted cell fractions was superior after negative compared to positive immunomagnetic selection (data not shown).

The expression of CK/VEGF, CK/VEGFR2 and CK/HIF-1α was investigated in PBMCs cytospins before and after immmunomagnetic separation. Figure 3BI, II shows that VEGF could be co-localized with VEGFR2 or HIF-1α in the same cell.

In order to further confirm that HIF-1α, VEGF and VEGFR2 were co-expressed in the same CTC and not in residual PBMCs, triple immunofluorence CK/VEGF/VEGFR2 and CK/VEGF/HIF-1α was performed in the same patients. Figure 3CI, II demonstrates that VEGF is co-expressed with VEGFR2 and HIF-1α in CTCs.

### VEGF mRNA expression in CTCs

To determine whether VEGF was produced by the CTC and not adsorbed from the plasma, RNA extracted from both the CTCs-enriched and the corresponding CTCs-depleted cell fractions were analyzed for VEGF using equal RNA amount of each fraction and quantitative real-time RT-PCR assay as previously described [24]. A 2^-ΔCt ^method [29] was used to examine the relative expression of VEGF in CTCs-enriched fraction compared to the CTCs-depleted fraction.

The results revealed that VEGF mRNA was overexpressed in four (40%) out of 10 examined patients (compared to corresponding fractions of normal blood donors) with a 3.1-, 8.3-, 11.5- and 25.3-fold increase in the CTCs-enriched cell fraction compared to the CTCs-depleted cell fraction. Double staining experiments with VEGF and A45/B-B3 antibodies confirmed the presence of intracytoplasmic VEGF in these four patients as well as in three additional patients (Table 2).

**Table 2:** VEGF mRNA expression in CTCs of patients with metastatic breast cancer

No Patients	Ct _(CTCs)_	Ct _(PBMCs)_	2^-ΔCt^
1	32,6	30,6	0,3
2	33,7	36,8	**8,3**
3	24,1	28,7	**25,3**
4	37,9	38,8	1,8
5	41,0	41,0	1,0
6	39,4	41,0	**3,1**
7	34,8	32,3	0,2
8	41,0	36,9	0,1
9	35,7	32,2	0,1
10	34,7	38,2	**11,5**

A 2^-ΔCt ^method was used to examine the relative expression of VEGF in CTCs-enriched fraction compared to the CTCs-depleted fraction. Equal RNA amount from each fraction was processed for RT-PCR and Real-Tme PCR. Ct is the Real-Time PCR sample threshold cycle and ΔCt is (Ct_(CTCs)_- Ct_(PBMCs)_), PBMCs are the Peripheral Blood Mononuclear Cells (the CTCs-depleted fraction).

## Discussion

Circulating and disseminated tumor cells derived from the primary tumor and possessing advantageous biological characteristics could acquire the capability of generating metastasis. CTCs have significant prognostic value for early and metastatic breast cancer patients [4,5,30]. These cells are very heterogenous and not fully characterized as yet [31,32]. VEGF is an important angiogenic factor for tumor vascularization. The expression of VEGF is under HIF-1α control, in hypoxia conditions [8,11]. In addition, focal adhesion kinase is also an upstream regulator of VEGF and HIF-1α expression [13,17]. We have recently reported that phosphorylated-FAK was expressed in CTCs of all CK-positive early breast cancer patients [18]. In the present study we examined the existence of angiogenic molecules in CTCs of metastatic breast cancer patients.

Initially, we evaluated the expression of VEGF in breast cancer cell lines. As shown in Figure 1, VEGF was expressed in the cytoplasm of all the examined breast cancer cells. Interestingly, a correlation was evident between VEGF expression and cell aggressiveness. Specifically, the SKBR3, MDA-MB-231 and MDA-MB-453 cell lines which are highly metastatic [25-27] expressed higher levels of VEGF compared to MCF7 and T47D which are estrogen receptor-positive and less aggressive cells. On the contrary, the total protein amount of HIF-1α didn't follow the same pattern of expression. This is probably attributed to the fact that cells were cultured under normal oxygen conditions. Moreover, HIF-1α is a transcription factor and it is possible that its activity relates to its nuclear distribution rather than the total intracellular amount of protein.

Since VEGF is related to the metastatic potential of cancer cells, we investigated VEGF expression in CTCs of 34 metastatic CK-positive breast cancer patients. The results of the current study revealed that VEGF was expressed in CTCs of 62% of the patients and the great majority (73%) of these cells were double positive (CK^+^VEGF^+^), implying the possible involvement of VEGF in the metastatic potential of these cells. Indeed, in the study by Mohammed *et al *[25-27], VEGF expression on breast cancer cells was associated with aggressive tumor biology as indicated by the significant correlation between VEGF expression, lymph node metastasis, distant metastasis and poorer survival.

In the present study, VEGF expression in CTCs was further confirmed by quantitative real-time RT-PCR in immunomagnetically separated CTCs. Indeed, CTCs from four out of 10 patients expressed both VEGF mRNA and VEGF protein. Immunofluorescence experiments further revealed VEGF protein expression in three additional patients who did not express VEGF mRNA. This discrepancy could be attributed to the different sensitivities of the used assays; alternatively, it is possible that this growth factor could be absorbed from the serum.

HIF-1α induces VEGF expression in cells under hypoxic conditions by binding to VEGF promoter [8,33]. Recent studies have shown that HIF-1α is increased in various carcinomas and its presence is associated with poor prognosis and resistance to therapy [16,34,35]. Focal adhesion kinase (FAK) is also important in promoting VEGF-induced tumor angiogenesis [13] whereas the expression of HIF-1α depends on FAK and PI-3 kinase activation in cancer cells [17]. In order to characterize the possible functional pathways leading to the observed VEGF expression in CTCs we investigated the expression of HIF-1α and pFAK in patients' CTCs. Double staining experiments revealed that HIF-1α was expressed in 76% of breast cancer patients and this expression was observed both in the cytoplasm and the nucleus although, in general, cytospin preparations are not the optimal material to study the intracellular distribution of molecules. The intracellular distribution reported in this study refers to what it was observed using confocal laser scanning microscopy (Figure 2B). Double (VEGF/HIF-1α) and triple (VEGF/HIF-1α/CK) staining immunofluoresence experiments after immunomagnetic enrichment of CTCs, revealed that these molecules can be co-localized in the same cell (Figure 3BII and 3CII). These findings denote a possible interaction between VEGF and HIF-1α in CTCs as it has already been shown in cancer cells [16]. In addition, our experiments revealed that FAK was phosphorylated in the CTCs of the majority (92%) of the examined CK-positive metastatic breast cancer patients. These results are in agreement with our previously published data from early breast cancer patients [18]. Furthermore, all patients with pFAK-positive CTCs also had detectable HIF-1α positive CTCs (Spearman's correlation analysis *P *< 0.001), suggesting the presence of a functional pathway leading to VEGF production.

VEGFR2 is the principal receptor through which VEGFs exert their mitogenic and chemotactic effects [8,36]. In breast cancer cell lines VEGF regulates cell growth and survival via an autocrine loop including the phosphorylation of Akt and ERK1/2 [15,37]. We have recently shown that Akt was strongly activated in CTCs both in patients with early and metastatic disease [19]; however, there are no data in the literature concerning the expression of VEGFR2 in CTCs. The results of the current study demonstrate that VEGFR2 was expressed in CTCs of 47% of breast cancer patients. It is interesting to note that the proportion of CTCs expressing VEGF or VEGFR2 in all the tested patients was almost identical (73% and 71%, respectively; Figure 1C); in addition, there was a statistically significant correlation between the number of CTCs expressing VEGF and VEGFR2 (*P *= 0.042). These data strongly suggest that patients' CTCs may both produce and consume VEGF through a putative autocrine mechanism. This assumption is further supported by the observed co-expression of these molecules (Figures 3BI and 3CI) in CTCs after immunomagnetic enrichment and double (VEGF/VEGFR2) or triple (VEGF/VEGFR/CK) staining experiments.

Our results also confirmed the heterogeneity of CTCs. Indeed, the molecules evaluated in the present study were not expressed in all examined CTCs, even not among those identified in the same patient. This may explain the fate and the metastatic potential of cells presenting such a phenotypic diversity.

## Conclusions

The data reported in the present study demonstrate that CTCs from patients with metastatic breast cancer express VEGF both at mRNA and protein level. The production of VEGF is probably under the regulation of HIF-1α and/or pFAK as suggested by the observed significant correlation between the expression of these molecules. Moreover, we have also demonstrated that VEGFR2 is also expressed on CTCs' membrane suggesting an autocrine loop involving VEGF and VEGFR2. The biologic significance of the presence of angiogenic molecules on CTCs is currently unknown. However, based on previous evidence, one might hypothesize that angiogenic pathways present on CTCs could result in evasion of apoptosis, enhancement of metastatic potential [10] and resistance to endocrine therapy [38]. In addition, the expression of angiogenic molecules on CTCs could be indicative of the tumor dependence on angiogenesis. Accordingly, anti-VEGF and/or anti-VEGFR2 targeting agents might have therapeutic implications in patients with VEGF- and VEGFR2-expressing CTCs.

## Abbreviations

CTCs: circulating tumor cells; HIF-1α: hypoxia inducible factor 1 alpha; HUVEC: human umbilical vein endothelial cells; PBMCs: peripheral blood mononuclear cells; PBS: Phosphate Buffered Saline solution; pFAK: phosphorylated-focal adhesion kinase; RT-PCR: reverse transcription-polymerase chain reaction; VEGF: vascular endothelial growth factor; VEGFR2: vascular endothelial growth factor receptor 2.

## Competing interests

The authors declare that they have no competing interests.

## Authors' contributions

GK participated in the design and coordination of the study. She performed immunofluoresence experiments as well as the immunomagnetic separations and the cell cultures. She performed reverse transcription (RT) reactions. She also drafted the manuscript. HM performed RNA extraction. VG performed some immunofluoresence experiments. MP collected all the clinicopathological characteristics of the patients. She also performed some immunofluoresence experiments. AS performed quantitative real-time RT-PCR. EL participated in the coordination of the study and helped to draft the manuscript. VG provided general support and participated in study design. He also drafted the manuscript, DM helped to draft the manuscript and SA participated in the coordination of the study and help drafted the manuscript.

## Supplementary Material

Additional file 1 A table listing the number of VEGF, VEGFR2 and HIF-1α-positive CTCs in patients with breast cancer. It is named as Supplementary Table S1. Click here for file

Additional file 2 A table listing the antibodies and the corresponding dilutions utilized in the present study. It is named as Supplementary Table S2. Click here for file

Additional file 3 A diagram presenting the quantification of VEGF, VEGFR2, HIF-1α and pFAK expression in breast cancer cell lines. It is named as Supplementary diagram. Click here for file
