# Supplementary material for: Hypoxia-inducible factor-1α and vascular endothelial growth factor expression in circulating tumor cells of breast cancer patients
Source: Breast Cancer Res. 2009 Nov 17;11(6):R84. doi: 10.1186/bcr2452 (PMC2815547; doi:10.1186/bcr2452)
Supplement: Additional file 1 — A table listing the number of VEGF, VEGFR2 and HIF-1α-positive CTCs in patients with breast cancer. It is named as Supplementary Table S1. [file bcr2452-S1.doc]

**Supplementary Table 1.** Antibodies and fluorochromes used in the present study

| Primary antibodies | Dilutions | Type |
| --- | --- | --- |
| A45-B/B3 | 1/50 | Anti-mouse |
| VEGF | 1/50 | Anti-goat |
| VEGF (Triple staining) | 1/50 | Anti-mouse |
| VEGFR2 | 1/50 | Anti-rabbit |
| HIF-1α | 1/50 | Αnti-rabbit |
| p-FAK | 1/25 | Anti-mouse |
| Pancytokeratin | 1/100 | Anti-rabbit |
| FITC | 1/100 | Anti-mouse |
| Rhodamine | 1/50 | Anti-goat |
| Alexa 555 | 1/200 | Anti-rabbit |
| Alexa 633 | 1/500 | Anti-mouse |
| Zenon IGg1 | 1/30 | Anti-mouse |
