# Supplementary material for: Hypoxia-inducible factor-1α and vascular endothelial growth factor expression in circulating tumor cells of breast cancer patients
Source: Breast Cancer Res. 2009 Nov 17;11(6):R84. doi: 10.1186/bcr2452 (PMC2815547; doi:10.1186/bcr2452)
Supplement: Additional file 2 — A table listing the antibodies and the corresponding dilutions utilized in the present study. It is named as Supplementary Table S2. [file bcr2452-S2.doc]

**Quantification of VEGF, VEGFR2, HIF-1α and pFAK expression in breast cancer cell lines.**

Quantification of VEGF, VEGFR2, HIF-1α and pFAK expression in each cell line was done compared to the corresponding actin.
