# Supplementary material for: Hypoxia-inducible factor-1α and vascular endothelial growth factor expression in circulating tumor cells of breast cancer patients
Source: Breast Cancer Res. 2009 Nov 17;11(6):R84. doi: 10.1186/bcr2452 (PMC2815547; doi:10.1186/bcr2452)
Supplement: Additional file 3 — A diagram presenting the quantification of VEGF, VEGFR2, HIF-1α and pFAK expression in breast cancer cell lines. It is named as Supplementary diagram. [file bcr2452-S3.doc]

**Supplementary Table 2.** Number of VEGF, VEGFR2 and HIF-1α-positive CTCs in patients with breast cancer.

|  | Patients (%) | | |
| --- | --- | --- | --- |
|  | VEGF | VEGFR2 | HIF-1α |
| No CTCs per sample | n=34 | n=34 | n=34 |
| 1 | 9 (26) | 6(18) | 11(32) |
| 2 | 6(18) | - | 4(12) |
| 3 | 1(3) | 3(9) | 4(12) |
| 4 | - | 2(6) | 2(6) |
| ≥ 5 | 5(15) | 5(15) | 5(15) |
